# Short-Term Intake of a Fructose-, Fat- and Cholesterol-Rich Diet Causes Hepatic Steatosis in Mice: Effect of Antibiotic Treatment

**DOI:** 10.3390/nu9091013

**Published:** 2017-09-14

**Authors:** Annette Brandt, Cheng Jun Jin, Katja Nolte, Cathrin Sellmann, Anna Janina Engstler, Ina Bergheim

**Affiliations:** 1 Department of Nutritional Sciences, Molecular Nutritional Science, University of Vienna, A-1090 Vienna, Austria; annette.brandt@univie.ac.at (A.B.); anna.engstler@univie.ac.at (A.J.E.); 2 Institute of Nutritional Sciences, SD Model Systems of Molecular Nutrition, Friedrich-Schiller-University Jena, D-07743 Jena, Germany; taiji-2002@hotmail.com (C.J.J.); KatjaNolte@gmx.net (K.N.); CathrinSellmann@gmx.de (C.S.)

**Keywords:** steatosis, NAFLD, antibiotics, intestinal permeability, microbiota

## Abstract

Intestinal microbiota and barrier functions seem to play an important role in the development of non-alcoholic fatty liver disease (NAFLD). However, whether these changes are an early event in the development of NAFLD or are primarily associated with later stages of the disease, has not yet been clarified. Using a pair-feeding model, we determined the effects of a short-term intake of a fat-, fructose- and cholesterol-rich diet (FFC) on the development of early hepatic steatosis and markers of intestinal barrier function in mice treated with and without non-resorbable antibiotics (AB). For four days, C57BL/6J mice were either pair-fed a control diet or a FFC diet ± AB (92 mg/kg body weight (BW) polymyxin B and 216 mg/kg BW neomycin). Hepatic steatosis and markers of inflammation, lipidperoxidation and intestinal barrier function were assessed. Lipid accumulation and early signs of inflammation found in the livers of FFC-fed mice were markedly attenuated in FFC + AB-fed animals. In FFC-fed mice the development of NAFLD was associated with a significant loss of tight junction proteins and an induction of matrix metalloproteinase-13 in the upper parts of the small intestine as well as significantly higher portal endotoxin levels and an induction of dependent signaling cascades in the liver. As expected, portal endotoxin levels and the expression of dependent signaling cascades in liver tissue were almost at the level of controls in FFC + AB-fed mice. However, FFC + AB-fed mice were also protected from the loss of zonula occludens-1 and partially of occludin protein in small intestine. Our data suggest that the development of early diet-induced hepatic steatosis in mice at least in part results from alterations of intestinal barrier function.

## 1. Introduction

With a prevalence ranging from ~2% to 44% in the general European population and ~24% among adults in Northern America, non-alcoholic fatty liver disease (NAFLD) is claimed to be the most common liver disease in the world by now [1,2]. NAFLD comprises a broad spectrum of diseases ranging from simple steatosis to steatohepatitis, cirrhosis and in some cases even to hepatocellular carcinoma [3]. However, in spite of intense research efforts, the molecular mechanisms underlying the development of NAFLD have not yet been clarified. Accordingly, universally accepted treatment options are also still limited and therapies targeting life-style and dietary patterns bearing high relapse rates are still the treatment strategies of choice [4].

Changes of intestinal microbiota composition and impairment of intestinal barrier function leading to an increased translocation of bacterial endotoxins and subsequently an activation of toll-like receptor (TLR)-dependent signaling cascades in the liver have repeatedly been discussed to be critical in the development of NAFLD (for overview see [5,6]). For example, it has been shown that the fecal microbiota composition and metabolic profile of children and adults with NAFLD are markedly altered from that of healthy controls [7,8] and that the prevalence of certain bacterial strains might be a risk factor in the development of non-alcoholic steatohepatitis and fibrosis [7]. Recently it has been reported that sub-therapeutic life-long intake of antibiotics leading to marked changes in fecal microbiota composition may even worsen insulin resistance and NAFLD in high fat diet-fed mice [9]. In contrast, in fructose-fed mice and rats, long-term concomitant treatment with therapeutic doses of antibiotics has been shown to protect animals against the onset of NAFLD or the metabolic syndrome [10,11]. In these studies, protection was associated with lower bacterial endotoxin levels in the portal vein and a less pronounced activation of the TLR-4-dependent signaling cascade in the liver [10,11]. Moreover, the results of animal and human studies suggest that a loss of tight junction proteins in the small intestine is frequently associated with increased intestinal permeability and portal endotoxin levels [12,13]. However, if the beneficial effects of a treatment with therapeutic doses of non-resorbable antibiotics during the onset of NAFLD are associated with changes of intestinal barrier function e.g., protection against the loss of tight junction proteins, or if these effects are only related to the elimination of bacteria from the small and large intestine, has not yet been fully clarified.

Starting from this background the aim of the present study was to determine if a short-term change of dietary pattern e.g., the intake of a fat-, fructose- and cholesterol-rich diet (FFC) for only four days, is sufficient to cause impairments of intestinal barrier function and the onset of NAFLD. Our study further aimed to determine if a treatment with therapeutic doses of non-resorbable antibiotics parallel to disease induction protects mice from changes to the intestinal barrier e.g., the loss of tight junction proteins, or if the protection associated with the antibiotic treatment is rather related to an elimination of bacteria in the gut.

## 2. Materials and Methods

### 2.1. Animals and Treatment

**Figure 1:**
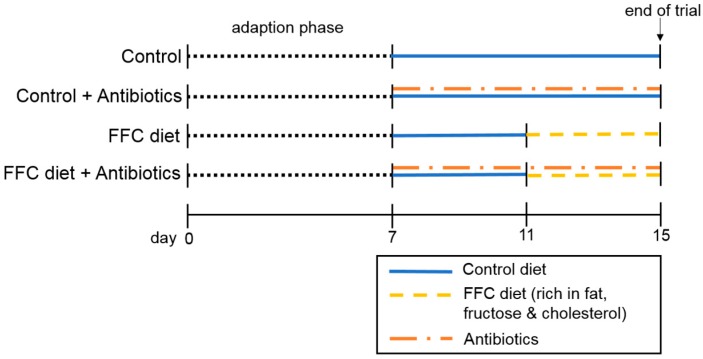
Summary of study design. After adapting mice for 7 days to the intake of a liquid diet followed by 4 days of pretreatment with the non-resorbable antibiotics (92 mg polymyxin B/kg body weight/day and 216 mg neomycin/kg body weight/day) or vehicle (=water) added to the liquid control diet, mice (*n* = 6–8/group) were either fed the liquid control diet or a diet rich in fat, fructose & cholesterol (FFC) ± antibiotics for another 4 days.

Female eight-week-old C57BL/6J mice (Janvier SAS, Le Genest-Saint-Isle, France) were housed in a specific-pathogen-free barrier facility accredited by the Association for Assessment and Accreditation of Laboratory Animal Care. All procedures were approved by the local institutional animal care and use committee (“Landesamt für Verbraucherschutz”, reference number: 02-021/14, Thuringia, Germany) and animals were handled in accordance to the European Convention for the Protection of Vertebrate Animals used for Experimental and Other Scientific Purposes. Weight gain was assessed weekly throughout the experiment. After adaption to the liquid diet for seven days, mice were randomly assigned to the following four different treatment groups (*n* = 6–8/group): control (C), C + antibiotics (C + AB), fat-, fructose- and cholesterol-rich diet (FFC), and FFC + AB. The composition of the diets fed are shown in Table S1 and have been published in detail previously [14]. The antibiotics used were a mixture of the non-resorbable antibiotics polymyxin (92 mg/kg body weight/day) and neomycin (216 mg/kg body weight/day; both Carl Roth, Karlsruhe, Germany). As also detailed in Figure 1, for the first four days of the experiment, all groups received a liquid standard diet (69E% carbohydrates, 12E% fat, 19E% protein; Ssniff, Soest, Germany) which was enriched with non-resorbable antibiotics dissolved in water in those groups assigned to be treated with AB e.g., C + AB and FFC + AB. Two groups were then switched from the control diet and the control diet + AB, respectively, to a liquid FFC diet (60E% carbohydrates, 25E% fat, 15E% protein with 50% wt/wt fructose and 0.16% wt/wt cholesterol; Ssniff, Soest, Germany) or FFC + AB for another four days, while the remaining control groups were fed the control diet or control diet + AB. Dosing of antibiotics was adapted from Bergheim et al. and Wagnerberger et al. [10,15]. In line with our previous studies, this dose of antibiotics reduced the number of colony-forming units in feces by ~90% compared to vehicle treated mice. Diet consumption was assessed daily and adjusted between groups so that all groups received the same amount of calories. At sacrifice, mice were anesthetized with a mix of ketamine (100 mg/kg body weight) and xylazine (16 mg/kg body weight) intraperitoneal. Blood was obtained from the portal vein. Portions of the liver and small intestine (duodenum and jejunum) were either frozen immediately, fixed in neutral-buffered formalin, frozen-fixed in optimum-cutting-temperature (OCT) mounting-media (Medite, Burgdorf, Germany) or stored in RNA*later*^®^ at −20 °C (Sigma-Aldrich, Steinheim, Germany).

### 2.2. Histological Evaluation of Liver Sections and Hepatic Lipid Accumulation

Liver histology was assessed in paraffin-embedded sections (4 µm) stained with hematoxylin and eosin (both Sigma-Aldrich, Steinheim, Germany) using the NAFLD activity score (NAS) as previously described [16]. Frozen sections of liver fixed in OCT (10 µm) were stained with Oil red O (Sigma-Aldrich, Steinheim, Germany) as described previously [17]. Representative pictures of both stainings were captured at a 200× magnification using a system incorporated in a microscope (Leica DM4000 B LED, Leica, Wetzlar, Germany). To determine the number of neutrophilic granulocytes in liver tissue, paraffin-embedded sections (4 µm) were stained using a commercially available Naphthol AS-D Chloracetate Esterase kit (Sigma-Aldrich, Steinheim, Germany). The number of neutrophils was quantified as detailed previously [13]. Hepatic triglycerides were extracted from whole liver tissue and measured as previously described [13].

### 2.3. Blood Parameters of Liver Damage, ELISA and Endotoxin Measurement

Plasma alanine transaminase (ALT) activity was determined using a colorimetric assay in a routine laboratory at University Hospital of Jena, Germany (Architect, Abbott GmbH & Co. KG, Wiesbaden, Germany). The protein concentration of plasminogen activator inhibitor-1 (PAI-1) in liver homogenate was determined using a commercially available mouse PAI-1 enzyme-linked immunosorbent assay (ELISA) kit (LOXO GmbH, Dossenheim, Germany) according to the manufacturers’ instructions. The endotoxin levels in portal plasma were measured with a commercially available limulus amebocyte lysate assay (Charles River, Ecully, France) as described previously [13].

### 2.4. Immunohistochemical Staining for 4-HNE Protein Adducts and iNOS Protein in the Liver As Well As 3-Nitrotyrosine Protein Adducts, MMP-13, Occludin and ZO-1 Protein in the Small Intestine

Paraffin-embedded liver sections (4 µm) were stained to determine the concentration of 4-hydroxynonenal (4-HNE) protein adducts and inducible nitric oxide synthase (iNOS) protein using polyclonal antibodies (4-HNE: (#H-1110) AG Scientific, San Diego, CA, USA; iNOS: (#PA3-030A) Thermo Fisher Scientific, Waltham, MA, USA) as described previously [10,18]. Additionally, paraffin-embedded sections of small intestine were stained for 3-nitrotyrosine (3-NT) protein adducts, matrix metalloproteinase-13 (MMP-13), occludin and zonula occludens-1 (ZO-1) protein (3-NT: (#sc-32757) Santa Cruz Biotechnology, Dallas, TX, USA; MMP-13: (#LS-B3168) LifeSpan BioSciences, Seattle, WA, USA; occludin and ZO-1 both Invitrogen (#71-1500, #61-7300), Carlsbad, CA, USA) as previously described [13,19], and for MMP-13 staining modified from Yang et al. [20]. In brief, tissue sections, treated with citrate buffer (3-NT, MMP-13) or protease (occludin and ZO-1) for antigen retrieval and blocked with bovine serum albumin solution (3-NT, MMP-13) were incubated with peroxidase-linked secondary antibodies and diaminobenzidine (Peroxidase Envision Kit, DAKO, Hamburg, Germany) to detect specific primary antibody binding to the target protein. For the assessment of the staining intensity of 3-NT, 4-HNE, iNOS, occludin and ZO-1, data were collected from eight pictures of each tissue section (200× for 3-NT, 4-HNE and iNOS, 400× for occludin and ZO-1) using a camera integrated in a microscope (Leica DM4000 B LED, Leica, Wetzlar, Germany).

### 2.5. RNA Isolation and Real-Time RT-PCR

RNA from the liver and small intestinal tissue was extracted (peqGOLD Trifast, Peqlab, Erlangen, Germany) and cDNA was synthesized using a reverse transcription system (Promega GmbH, Madison, WI, USA). Expression of acetyl-CoA carboxylase (*ACC*), fatty acid synthase (*FASN*), interleukin-1*β* (*Il-1β*), interleukin-6 (*Il-6*), toll-like receptor-4 (*TLR-4*), stearoyl-CoA desaturase-1 (*SCD1*), sterol regulatory element-binding protein-1c (*SREBP-1c*), myeloid differentiation primary response gene 88 (*MyD88*) mRNA in the liver and matrix metalloproteinase-9 (*MMP-9*) and *MMP-13* mRNA in the small intestine were measured using real-time polymerase chain reaction (PCR) as detailed previously [14]. Primer sequences are shown in Table 1. For determination of the amount of target genes, the comparative C_T_-method was used and results were normalized to the endogenous reference 18S and relative to a calibrator (2^−ΔΔ*C*t^).

### 2.6. Western Blot

Snap frozen tissue from the small intestine was homogenized in a radio-immunoprecipitation assay (RIPA) lysis buffer (20 mM 3-(*N*-morpholino)propanesulfonic acid (MOPS), 150 mM NaCl, 1 mM ethylenediaminetetraacetic acid (EDTA), 1% Nonidet P-40 and 0.1% sodium dodecyl sulfate (SDS)) containing protease and phosphatase inhibitor cocktails (both Sigma-Aldrich, Steinheim, Germany) to isolate protein [13,21]. After measuring the protein concentration of each sample using the Bradford protein assay (Bio-Rad Protein Assay Kit II, Bio-Rad Laboratories, Hercules, CA, USA), protein lysates (30 µg protein/well) were separated in a 12% SDS-polyacrylamide gel and transferred to an Immun-Blot^®^-polyvinylidene difluoride membrane (Bio-Rad Laboratories, Hercules, CA, USA) [13]. As some of the tissue samples showed signs of degradation, we were only able to include four samples per group for the detection of the tight junction proteins junctional adhesion molecule-1 (JAM-1) and claudin-2. After incubating blots with primary antibodies against claudin-2, JAM-1 (both abcam (#ab52647, #ab125293), Cambridge, UK) or β-actin (Cell Signaling (#4970), Danvers, MA, USA) and respective secondary antibodies (Cell Signaling, Danvers, MA, USA), protein bands were detected using a luminol-based enhanced chemiluminiescence horseradish peroxidase (HRP) substrate (Super Signal West Dura kit, Thermo Fisher Scientific, Waltham, MA, USA). Densitometric analysis was performed using the ChemiDoc MP System (Bio-Rad Laboratories, Hercules, CA, USA) as detailed before [13].

### 2.7. Statistical Analysis

All data are presented as mean ± standard error of mean (SEM). Outliers were identified using Grubb’s test. The homogeneity of variances was tested with Bartlett’s test and in cases of unequal variances the raw data were log-transformed before performing the two-factorial analysis of variance (ANOVA) to determine the statistical differences between groups (Graph Pad Prism Software 6, San Diego, CA, USA). A *p* value < 0.05 was considered to be significant.

## 3. Results

### 3.1. Body Weight, Liver Steatosis and Inflammatory Markers in Liver

**Figure 2:**
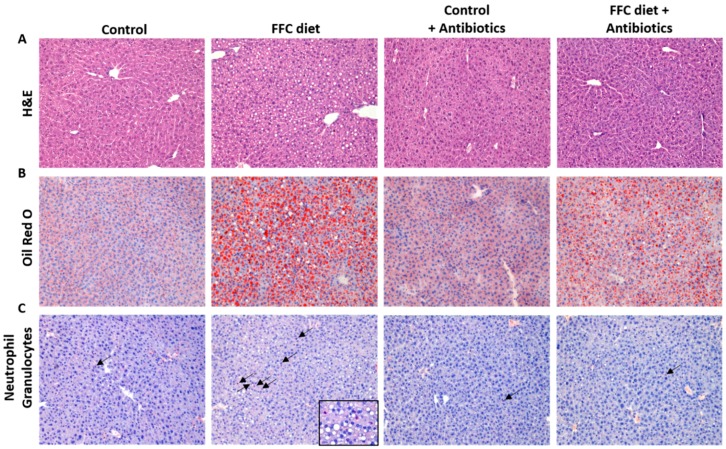

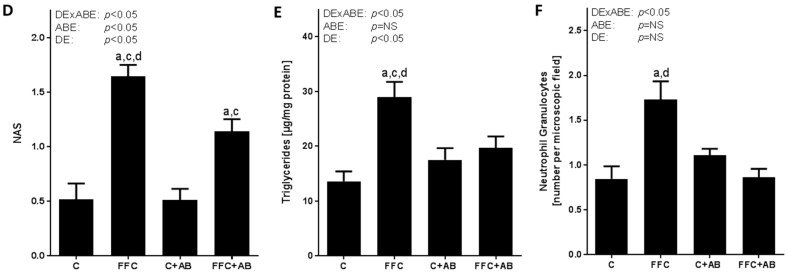
Effect of the short-term feeding of a FFC or control diet ± oral antibiotic treatment on liver. Representative pictures of (**A**) heamatoxylin and eosin staining (200×); (**B**) Oil Red O staining (200×) and (**C**) neutrophilic granulocytes (200×; arrows are indicators for neutrophilic granulocytes; FFC section 630×) of liver sections; (**D**) Evaluation of liver damage using NAS and (**E**) quantification of hepatic triglyceride accumulation; (**F**) Number of neutrophilic granulocytes in liver. ABE: antibiotic effect; C: control diet; C + AB: control diet and oral treatment with antibiotics; DE: diet effect; DE × ABE: interaction between diet and antibiotics; FFC: fat-, fructose- and cholesterol-rich diet; FFC + AB: fat-, fructose- and cholesterol-rich diet and oral treatment with antibiotics; NAS: NAFLD activity score; NS: not significant. ^a^ *p* < 0.05 compared with mice fed a control diet; ^c^ *p* < 0.05 compared with mice fed a control diet treated with antibiotics; ^d^ *p* < 0.05 compared with mice fed a FFC diet treated with antibiotics.

Despite being fed the different diets for only four days and a similar total caloric intake between groups and no significant differences in body weight gain (see Table 2), the livers of the FFC-fed mice displayed marked signs of beginning NAFLD. Indeed, NAS, used to determine liver histology and triglyceride concentrations, as well as the number of neutrophils in the liver tissue, were all significantly higher in the FFC-fed mice when compared to controls (*p* < 0.05 for all parameters when compared to controls) (see Figure 2). In contrast, NAS was significantly lower in the livers of FFC + AB-fed mice when compared to mice only fed the FFC diet; however, NAS was still significantly higher than in both control groups, while the hepatic triglyceride concentration and average number of neutrophils were at the level of the controls (see Figure 2). Expression of *Il-6* and *Il-1β* mRNA and the PAI-1 protein in liver tissue did not differ between the controls and FFC-fed mice. However, PAI-1 protein levels were significantly lower in the livers of FFC + AB-fed and C + AB-fed mice when compared to FFC-fed mice, while expression of *Il-1β* in the liver was only significantly lower in the livers of C + AB-fed mice than in FFC-fed animals (see Table 3). Liver weight and liver to body weight ratio were also significantly higher in the two FFC-fed groups regardless of additional treatment and did not differ between FFC-groups. As the mice only showed signs of a beginning NAFLD, ALT plasma activity was still at the level of the controls (see Table 2).

### 3.2. Markers of Lipogenesis in Liver

The expression of *SREBP-1c* mRNA did not differ between the FFC-fed groups and was markedly higher than in the livers of both control groups (*p* < 0.05 for FFC and FFC + AB vs. C + AB and *p* = 0.075 for FFC and *p* = 0.072 for FFC + AB vs. C). The expression of *ACC* was significantly higher in the livers of FFC- and C + AB-fed mice (*p* < 0.05 for both) and induced by trend in FFC + AB-fed mice (*p* = 0.091) when compared to the controls receiving vehicle. While expression of *FASN* in the liver was similar between groups, expression of *SCD1* mRNA was significantly higher in the livers of FFC-fed mice when compared to both control groups. Similar differences were also found when comparing hepatic *SCD1* expression between FFC + AB-fed mice and controls without antibiotic treatment (see Table 3).

### 3.3. Markers of TLR-4 Signaling As Well As Lipidperoxidation in Liver Tissue

**Figure 3:**
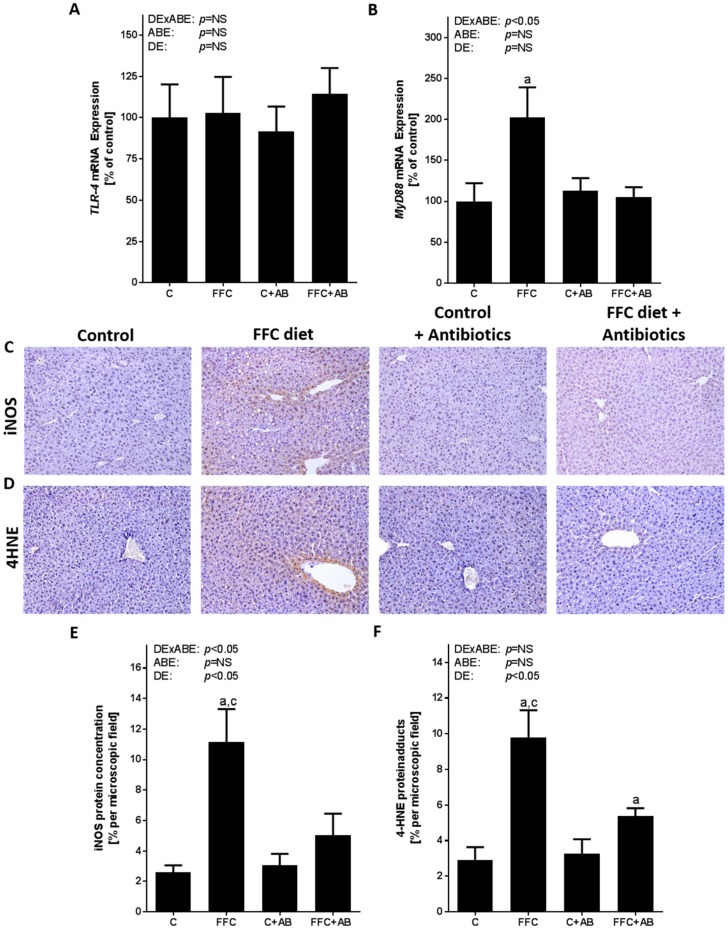
Effect of the short-term feeding of a FFC or control diet ± oral antibiotic treatment on markers of TLR-4 signaling cascade and lipidperoxidation in liver tissue. (**A**) *TLR-4* and (**B**) *MyD88* mRNA expression in liver tissue; (**C**,**D**) Representative pictures (200×) and (**E**,**F**) densitometric analysis of iNOS and 4-HNE protein adduct staining in liver tissue. 4-HNE: 4-hydroxynonenal; ABE: antibiotic effect; C: control diet; C + AB: control diet and oral treatment with antibiotics; DE: diet effect, DE × ABE: interaction between diet and antibiotics; FFC: fat-, fructose- and cholesterol-rich diet; FFC + AB: fat-, fructose- and cholesterol-rich diet and oral treatment with antibiotics; iNOS: inducible nitric oxide synthase; MyD88: myeloid differentiation primary response gene 88; NS: not significant; TLR-4: toll-like receptor-4. ^a^ *p* < 0.05 compared with mice fed a control diet; ^c^ *p* < 0.05 compared with mice fed a control diet treated with antibiotics.

The expression of *TLR-4* mRNA in the liver tissue was similar between groups. In contrast, the expression of *MyD88* mRNA was significantly higher in the livers of the FFC-fed mice than in the controls (*p* < 0.05), while expression of *MyD88* mRNA in the livers of the FFC + AB-fed mice did not differ from that of both control groups (NS for C vs. FFC + AB and C + AB vs. FFC + AB) (see Figure 3).

The increased level of *MyD88* mRNA expression in the liver tissue were associated with significantly higher iNOS and 4-HNE protein adduct levels (+4.3- and +3.4-fold compared to C, respectively) in the livers of FFC-fed mice when compared with both control groups. Similar differences were not found when iNOS protein levels were compared between the controls and FFC + AB-fed mice. However, the 4-HNE concentration was significantly higher, by ~1.8 times, in the livers of FFC + AB-fed mice when compared to the control group without AB-treatment (see Figure 3).

### 3.4. Markers of Intestinal Barrier Function and Inflammation As Well As Lipidperoxidation in the Small Intestine and Portal Vein

**Figure 4:**
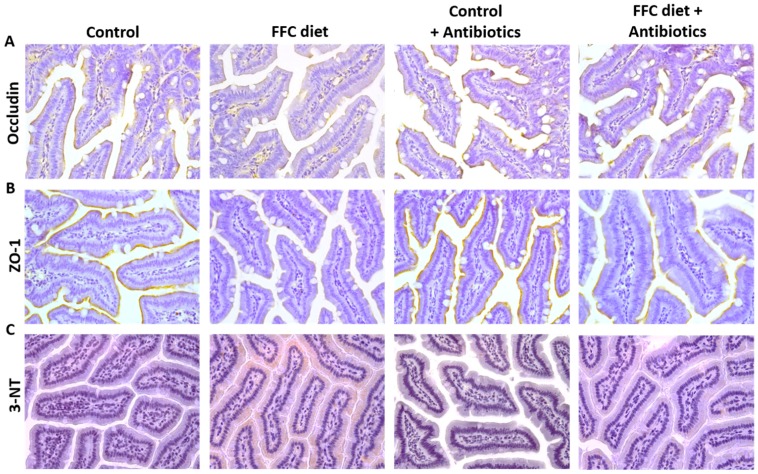

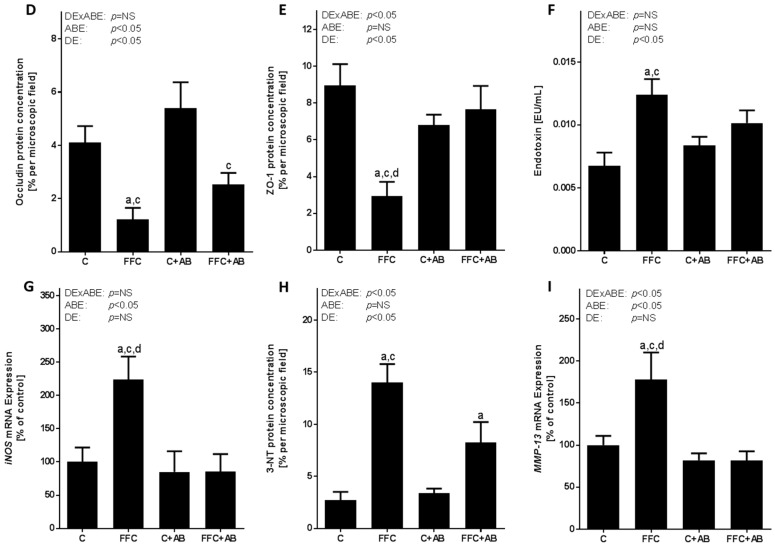
Effect of the short-term feeding of a FFC or control diet ± oral antibiotic treatment on markers of intestinal barrier function and *iNOS* mRNA expression as well as 3-NT protein adducts and *MMP-13* mRNA expression in small intestine. Representative pictures of (**A**) occludin; (**B**) ZO-1 and (**C**) 3-NT staining (400×) in small intestine and densitometric analysis of (**D**) occludin and (**E**) ZO-1 protein staining in small intestine and (**F**) endotoxin concentration in plasma of portal vein; (**G**) *iNOS* mRNA expression; (**H**) densitometric analysis of 3-NT protein adduct staining and (**I**) *MMP-13* mRNA expression in small intestine. 3-NT: 3-nitrotyrosine; ABE: antibiotic effect; C: control diet; C + AB: control diet and oral treatment with antibiotics; DE: diet effect; DE × ABE: interaction between diet and antibiotics; FFC: fat-, fructose- and cholesterol-rich diet; FFC + AB: fat-, fructose- and cholesterol-rich diet and oral treatment with antibiotics; iNOS: inducible nitric oxide-synthase; MMP-13: matrix metalloproteinase-13; NS: not significant; ZO-1: zonula occludens-1. ^a^ *p* < 0.05 compared with mice fed a control diet; ^c^ *p* < 0.05 compared with mice fed a control diet treated with antibiotics; ^d^ *p* < 0.05 compared with mice fed a FFC diet treated with antibiotics.

The endotoxin concentration was significantly higher in the portal plasma obtained from FFC-fed mice in comparison to both control groups, while similar differences were not found when comparing the control groups and FFC + AB-fed mice (see Figure 4). Protein concentrations of the tight junction proteins occludin and ZO-1 in the small intestine were significantly lower in the FFC-fed mice compared to both control groups (*p* < 0.05). A similar loss of tight junction proteins was not found in small intestine of the FFC + AB-fed mice for ZO-1. Indeed, the protein levels of ZO-1 were significantly higher in the small intestine of FFC + AB-fed mice when compared with mice only fed the FFC diet (*p* < 0.05 for FFC + AB vs. FFC). Occludin protein levels in FFC + AB fed mice were not different from those of the control; however, the levels were still lower than those of C + AB-fed animals (see Figure 4). As the quality of the lysates was only sufficient in *n* = 4 samples per group, no statistical analysis was performed for the protein levels of the tight junction protein claudin-2 and JAM-1 determined by Western blot (Figure S1). Furthermore, in the FFC-fed mice the expression of *iNOS* mRNA in the small intestinal tissue was significantly higher than in the controls. A similar induction of *iNOS* mRNA expression was not found in the small intestinal tissue of the FFC + AB-fed animals (see Figure 4). The protein concentration of 3-NT in the small intestine was also significantly higher in FFC-fed mice compared to both control groups. In contrast, the 3-NT levels in the small intestinal tissue of FFC + AB-fed mice were only significantly higher in comparison to C (see Figure 4). In line with previous findings of our group [13], the loss of tight junction proteins in the small intestine of the FFC-fed mice went along with a significant induction of the expression of *MMP-13* mRNA in this part of the intestine (see Figure 4). In addition, representative immunohistochemical staining of MMP-13 protein in the small intestinal tissue are shown in Figure S1. Similar differences between groups were not found for *MMP-9* mRNA expression (see Figure S1).

## 4. Discussion

### 4.1. Short-Term Isocalcoric Intake of a Fat-, Fructose- and Cholesterol-Rich Diet Leads to Alterations of the Markers of Intestinal Permeability and the Onset of NAFLD

Despite intense research efforts into the molecular mechanisms involved in the onset of NAFLD, this is still not fully understood and universally accepted prevention strategies are not yet available. The results of several epidemiological, clinical and animal based studies suggest that general over-nutrition, but also changes in dietary patterns such as a switch from a low fat, polysaccharide- and fiber-rich diet to a fat and sugar dominated diet or vice versa may lead to changes of intestinal microbiota composition and/or intestinal barrier function [22,23] and subsequently the development of NAFLD [24]. However, whether the changes in intestinal barrier function and the increased translocation of bacterial endotoxins are early events associated with the onset of NAFLD, and whether the intestinal microbiota is involved in this has not yet been fully clarified. Here, using a mouse model, we were able to show that alterations in intestinal barrier function, previously shown to be associated with the development of NAFLD, induced by a chronic intake of diets rich in fat, fructose and cholesterol or a single macronutrient-rich diets [13,25,26] are already present after a short-term switch of control to a fat-, fructose- and cholesterol-rich diet. In line with the findings regarding the chronic intake of macronutrient-rich diets [13,25,26], FFC-fed mice showed significantly increased *iNOS* mRNA expression, 3-NT protein adduct levels and *MMP-13* mRNA expression, as well as a loss of tight junction proteins in the small intestine and an increase in bacterial endotoxin levels in the portal plasma. Indeed, it has been suggested that the induction of *iNOS* expression and subsequently of NO synthesis in intestinal cells adds to the enhanced turn-over of tight junction proteins [27]. Furthermore, the results of other studies suggest that MMPs like MMP-13 may be involved in the degradation of tight junction proteins [28,29,30]. Tang et al. (2009) have shown that the inhibition of iNOS has protective effects on impaired intestinal permeability in settings of alcohol-induced intestinal barrier damage [31]. Results of in vitro studies further suggest that PI3K/AKT-dependent signaling cascades might be involved in the induction of iNOS and subsequently the loss of tight junction proteins [32,33]. Furthermore, Vandenbroucke et al. were able to demonstrate that MMP-13-/-mice are protected against LPS-induced impairment of intestinal barrier function [30].

Alterations found at the level of the small intestine in FFC-fed mice were associated with early signs of liver disease, e.g., fat accumulation. The increased fat accumulation was associated with an induction of genes involved in lipogenesis as well as with increased number of neutrophils [25,34]. However, as the mice only displayed the early signs of liver disease, the ALT activity in the plasma did not yet differ between groups. Moreover, the expression of *MyD88* mRNA and iNOS protein, as well as 4-HNE protein adducts in the liver—which have been previously shown to be highly dependent on the endotoxin-dependent activation of the TLR-4 signaling cascade in settings of diet-induced NAFLD [15,18,19]—were also significantly higher in the FFC-fed mice. The lack of a significant induction of *TLR-4* mRNA expression might have resulted from the short duration of the treatment, but also from the fact that proteins such as TLR-4 are not solely regulated by mRNA expression, but also through the altering of their half-life [35]. In previous studies targeting the TLR-4-dependent signaling cascade and tumor necrosis factor-α signaling, we showed that even during the onset and very early stage of NAFLD, e.g., steatosis without inflammation, disrupting the TLR-4 signaling cascades was associated with lower fat accumulation [18,19,36], suggesting that an activation of these cascades may already be critical to the development of steatosis. However, future studies will have to address this in more detail. Our findings are in line with the studies of others in rodents and humans showing that the short-term intake of a high fat diet is associated with the development of steatosis and in rodents an activation of TLR-4-dependent signaling cascades in the liver [37,38,39]. Taken together, the results of the present study suggest that even in settings of “normal” caloric intake a short-term change of dietary composition towards a more “unhealthy” dietary pattern e.g., a fat-, sugar- and cholesterol-rich diet can lead to a loss of tight junction proteins associated with increased lipidperoxidation and *MMP-13* levels in the small intestine, as well as elevated portal bacterial endotoxin levels and an activation of dependent signaling cascades in the liver and the development of hepatic steatosis.

### 4.2. Antibiotic Treatment Not Only Diminishes the Loss of Tight Junction Proteins and the Induction of iNOS in the Small Intestine and the Increase of Portal Endotoxin Levels, But Also the Accumulation of Triglycerides in the Liver

It has been shown previously that oral treatment with antibiotics over an extend period of time, e.g., several weeks, protects rodents at least in part from the development of NAFLD induced through various diets [10,15,40]. Furthermore, the results of human studies suggest that treatment with antibiotics may improve liver status in patients with NAFLD [41]. In the present study, antibiotic treatment protected mice from the early signs of diet-induced NAFLD, e.g., hepatic fat accumulation and an increase in the number of neutrophils, was markedly attenuated. The effects of the antibiotics were associated with decreased bacterial endotoxin level in portal plasma and no induction of dependent signaling cascades in the liver e.g., *MyD88* and iNOS. These findings are in line with previous studies treating rodents fed an ‘NAFLD’-diet with antibiotics [15,40,42,43,44]. Furthermore, the protective effects of the treatment with antibiotics on these early signs of hepatic steatosis were associated with a protection against the loss of the tight junction protein ZO-1 in the small intestine. These data further support the hypothesis that changes in the intestinal microbiota might be critical in the development of intestinal barrier dysfunction in patients with NAFLD (for overview see [45]). Furthermore, Douhara et al. demonstrated that the loss of tight junction proteins and higher intestinal permeability found in rats with hepatic fibrosis was almost completely ameliorated when the animals were treated with antibiotics [43]. In addition, it has been shown that an increased prevalence of certain bacterial strains such as *Akkermansia muciniphila* in the gut of rodents is associated with alterations of mucosa composition and tight junction proteins in the intestine as well as portal endotoxin levels and an improvement of glucose tolerance [46,47,48]. However, in these studies, different animal models were used and the animals were treated for a markedly longer period of time.

In line with our previous findings [13] and those of others in models of alcohol- and endotoxin-induced intestinal barrier dysfunction [31,49] as well as animal models of necrotizing enterocolitis (for an overview see [50]), in the present study, protection against the loss of tight junction proteins was associated with protection against the induction of *MMP-13* and *iNOS* mRNA expression in intestinal tissue. As already detailed above, MMPs and a dysregulation of NO production have been shown before to be involved in the weakening of intestinal barrier function [13,30,51,52]. However, the molecular mechanisms involved, e.g., how diet and/or bacteria or products derived from bacterial metabolism in settings of diet-induced NAFLD induce iNOS and probably subsequently MMP-13 remains to be determined in future studies. Still, our data lend further support to the hypothesis that a dysregulation of NO formation in the gut might be involved in the development of intestinal barrier dysfunction associated with the development of NAFLD.

Interestingly, despite significantly lower hepatic triglyceride levels in the FFC + AB-fed mice, the changes in the expression of the genes involved in hepatic lipogenesis found in the mice fed the fat-, fructose- and cholesterol-rich diet were not affected by the treatment with antibiotics. These alterations might have resulted more directly from the changes in dietary composition e.g., the increase in saturated fatty acids, fructose and cholesterol than the effect of antibiotics on intestinal microbiota and the translocation of bacterial endotoxins. In support of this hypothesis it has been shown before that an increased intake of fat but also of fructose is associated with an induction of hepatic lipid metabolism [34,53]. Indeed, the induction of *ACC* and *FASN* expression in the livers of the control mice treated with antibiotics—found in the present study—suggests that alterations of the microbiota may also influence the markers of lipogenesis. In support of this hypothesis, Singh et al. demonstrated that in TLR-5 deficient mice, showing altered microbiota, the markers of lipogenesis such as *SREBP-1c*, *ACC*, *FASN* and *SCD1* in liver tissue were also altered [54]. In this study, it was further concluded that SCD1 in liver tissue may be regulated through metabolites derived from intestinal microbiota as SCD1 converts short-chain fatty acids to mono-unsaturated fatty acids [54]. However, the apparent discrepancy of the expression of markers of lipogenesis and hepatic triglycerides suggests that other factors might have been involved in the lower triglyceride levels found in the liver of FFC + AB-fed mice. Indeed, the results of our own studies suggest that bacterial endotoxins through tumor necrosis factor-α and PAI-1-dependent signaling cascades might alter microsomal triglyceride transfer proteins, thereby modulating hepatic lipid export through very low-density lipoproteins [13,21]. Whether similar effects were involved in the alterations and effects found in the present study, remains to be determined.

## 5. Conclusions

In summary, the results of our study suggest that even a short-term intake of a fat-, fructose- and cholesterol-rich diet is sufficient to induce a loss of tight junction proteins in the small intestine and an increased translocation of bacterial endotoxins, and that oral treatment with antibiotics can prevent or at least in part attenuate these alterations. If this is the direct effect of the antibiotics or whether it is mediated through the loss and/or changes to the intestinal microbiota remains to be determined. It also remains to be determined whether the effects on intestinal barrier function found in the present study are sustainable and which are the underlying molecular mechanisms. However, our results suggest that the short-term intake of a fat-, fructose- and cholesterol-rich diet is sufficient to induce early signs of NAFLD in mice, and also that these are markedly attenuated by treatment with antibiotics. Future studies will have to determine whether similar effects of antibiotics are also found in humans when used as therapies for patients or for animal models with pre-existing NAFLD.

## Figures and Tables

**Table 1:** Primer sequences.

Gene	Forward (5′–3′)	Reverse (5′–3′)	Accession Number
*18S*	GTA ACC CGT TGA ACC CCA TT	CCA TCC AAT CGG TAG TAG CG	NR_003278
*ACC*	CTT CCT CCT GAT CAG CAA CTC T	CGT GAG TTT TCC CAA AAT AAG C	NM_133904
*FASN*	TCT GGG CCA ACC TCA TTG GT	GAA GCT GGG GGT CCA TTG TG	NM_007988
*Il-1β*	TGG CTG TGG AGA AGC TGT GG	GTC CGA CAG CAC GAG GCT TT	NM_008361
*Il-6*	CCA CGG CCT TCC CTA CTT CA	TGC AAG TGC ATC ATC GTT GTT C	NM_001314054
*iNOS*	CCC CTG GAA GTT TCT CTT CAA AGT C	GAT TCT GGA ACA TTC TGT GCT GTC C	NM_010927
*MMP-13*	AGA AGT GTG ACC CAG CCC TA	GCG CAA GAA GAA TCT GTC TTT	NM_008607
*MMP-9*	TGG TCT TCC CCA AAG ACC TG	GCG GTA CAA GTA TGC CTC TG	NM_013599
*MyD88*	CAA AAG TGG GGT GCC TTT GC	AAA TCC ACA GTG CCC CCA GA	NM_010851
*SCD1*	CCG ATA AAA GGG GGC TGA GG	TGC TGA GAT CGA GCG TGG AC	NM_009127
*SREBP-1c*	ACC GGC TAC TGC TGG ACT GC	AGA GCA AGA GGG TGC CAT CG	NM_001313979
*TLR-4*	AGC CAT TGC TGC CAA CAT CA	GCT GCC TCA GCA GGG ACT TC	NM_021297

ACC: acetyl-CoA carboxylase; FASN: fatty acid synthase; Il: interleukin; iNOS: inducible nitric oxide synthase; MMP: matrix metalloproteinase; MyD88: myeloid differentiation primary response gene 88; TLR: toll-like receptor; SCD1: stearoyl-CoA desaturase-1; SREBP-1c: sterol regulatory element-binding protein-1c.

**Table 2:** Caloric intake, body weight gain and markers of liver health of mice fed short-term a FFC or control diet ± antibiotic treatment.

Parameter	Groups				*p*-Value		
	C	FFC	C + AB	FFC + AB	DE × ABE	ABE	DE
Caloric intake [kcal/mouse/day]	9.9 ± 0.0	10.3 ± 0.2	9.7 ± 0.0	9.9 ± 0.2	NS	NS	NS
Body weight [g]	19.1 ± 0.3	19.9 ± 0.4	19.5 ± 0.3	19.6 ± 0.3	NS	NS	NS
Weight gain [g]	0.8 ± 0.2	0.8 ± 0.1	0.9 ± 0.2	1.3 ± 0.2	NS	NS	NS
Liver weight [g]	0.9 ± 0.02	1.0 ± 0.02 ^a,c^	0.9 ± 0.01	1.0 ± 0.02 ^a,c^	NS	NS	<0.05
Liver:body weight ratio [%]	4.6 ± 0.07	4.9 ± 0.08 ^a,c^	4.5 ± 0.05	5.0 ± 0.08 ^a,c^	NS	NS	<0.05
Plasma ALT [U/I]	19.6 ± 2.8	14.0 ± 0.7	14.9 ± 1.0	15.5 ± 1.4	NS	NS	NS

Values are means ± standard error of means. ABE: antibiotic effect; ALT: alanine transaminase; C: control diet; C + AB: control diet and oral treatment with antibiotics; DE: diet effect; DE × ABE: interaction between diet and antibiotics; FFC: fat-, fructose- and cholesterol-rich diet; FFC + AB: fat-, fructose- and cholesterol-rich diet and oral treatment with antibiotics; NS: not significant. ^a^ *p* < 0.05 compared with mice fed a control diet; ^c^ *p* < 0.05 compared with mice fed a control diet treated with antibiotics.

**Table 3:** Effect of the short-term feeding of a FFC or control diet ± antibiotic treatment on markers of inflammation and lipogenesis in liver tissue.

Parameter	Groups				*p*-Value		
	C	FFC	C + AB	FFC + AB	DE × ABE	ABE	DE
*Il-6* mRNA	100 ± 14	198 ± 45	133 ± 28	109 ± 14	NS	NS	NS
*Il-1β* mRNA	100 ± 12	127 ± 19 ^c^	61.3 ± 12	96.4 ± 16	NS	NS	<0.05
PAI-1 protein	100 ± 6	123 ± 10 ^c,d^	75.1 ± 4	65.1 ± 13 ^a^	NS	<0.05	NS
*SREBP-1c* mRNA	100 ± 20	182 ± 10 ^c^	88.0 ± 15	193 ± 25 ^c^	NS	NS	<0.05
*ACC* mRNA	100 ± 26	298 ± 59 ^a^	270 ± 50 ^a^	243 ± 40	<0.05	NS	NS
*FASN* mRNA	100 ± 24	146 ± 12	228 ± 46	258 ± 95	NS	NS	NS
*SCD1* mRNA	100 ± 21	429 ± 111 ^a,c^	109 ± 23	286 ± 65 ^a^	NS	NS	<0.05

Values are means ± standard error of means and are shown as % of control. ABE: antibiotic effect; ACC: acetyl-CoA carboxylase; C: control diet; C + AB: control diet and oral treatment with antibiotics; DE: diet effect; DE × ABE: interaction between diet and antibiotics; FFC: fat-, fructose- and cholesterol-rich diet; FFC + AB: fat-, fructose- and cholesterol-rich diet and oral treatment with antibiotics; FASN: fatty acid synthase; Il-1β: interleukin-1β; Il-6: interleukin-6; NS: not significant; PAI-1: plasminogen activator inhibitor-1; SCD1: stearoyl-CoA desaturase-1; SREBP-1c: sterol regulatory element-binding protein-1c. ^a^ *p* < 0.05 compared with mice fed a control diet; ^c^ *p* < 0.05 compared with mice fed a control diet treated with antibiotics; ^d^ *p* < 0.05 compared with mice fed an FFC diet treated with antibiotics.

## Supplementary Materials

The following are available online at www.mdpi.com/2072-6643/9/9/1013/s1, Table S1: Nutrient composition of control diet and fat-, fructose- and cholesterol-rich diet (FFC) (both Ssniff, Germany), Figure S1: Effect of a short-term feeding of a FFC or control diet ± oral antibiotic treatment on different tight junction proteins and MMPs in small intestine.
