# Supplementary material for: Short-Term Intake of a Fructose-, Fat- and Cholesterol-Rich Diet Causes Hepatic Steatosis in Mice: Effect of Antibiotic Treatment
Source: Nutrients. 2017 Sep 14;9(9):1013. doi: 10.3390/nu9091013 (PMC5622773; doi:10.3390/nu9091013)
Supplement: Supplementary file 1 [file nutrients-09-01013-s001.zip › nutrients-206726-supplementary.pdf]

Table S1. Nutrient composition of control diet and fat-, fructose- and cholesterol-rich diet (FFC) (both Ssniff, Germany)

		Control diet	FFC
**Crude protein, CP**	% wt/wt	17.4	16.0
**Crude fat, CL**	% wt/wt	5.1*	11.8*
**Crude fibre, CF**	% wt/wt	5.0	2.0
**Crude ash**	% wt/wt	4.1	4.2
**Corn Starch**	% wt/wt	34.6	5.0
**Sucrose**	% wt/wt	11.0	-
**Glucose**	% wt/wt	-	5.0
**Fructose**	% wt/wt	-	50.0
**Cholesterol**	% wt/wt	-	0.16
**L-Lysine**	% wt/wt	1.43	1.32
**L-Methionine + L-Cystein**	% wt/wt	1.00	0.96
**L-Threonine**	% wt/wt	0.75	0.69
**Calcium**	% wt/wt	0.78	0.77
**Phosphorus**	% wt/wt	0.48	0.47
**Sodium**	% wt/wt	0.23	0.30
**Vitamin A (retinol acetate)**	IU/kg	15000	15000
**Vitamin D_3 (cholecalciferol)**	IU/kg	1500	1500
**Vitamin E (α-tocopherol acetate)**	mg/kg	150	150
**Fatty acids**			
**C4:0**	% wt/wt	-	0.44
**C6:0**	% wt/wt	-	0.29
**C8:0**	% wt/wt	-	0.16
**C10:0**	% wt/wt	-	0.35
**C12:0**	% wt/wt	-	0.40
**C14:0**	% wt/wt	0.02	1.23
**C16:0**	% wt/wt	0.57	3.10
**C18:0**	% wt/wt	0.18	1.14
**C18:1 (n-9)**	% wt/wt	1.28	2.58
**C18:2 (n-6)**	% wt/wt	2.65	0.21
**C18:3 (n-3)**	% wt/wt	0.29	0.06
**Metabolizable energy (ME)**	kcal/kg	3752	4254
**CP**	kcal%	19	15
**CL**	kcal%	12	25
**Carbohydrate, CHO**	kcal%	69	60

*Fat source in control diet is soybean oil, fat source in FFC diet is butter fat. Values for crude fat are not corrected for water content of fat source.

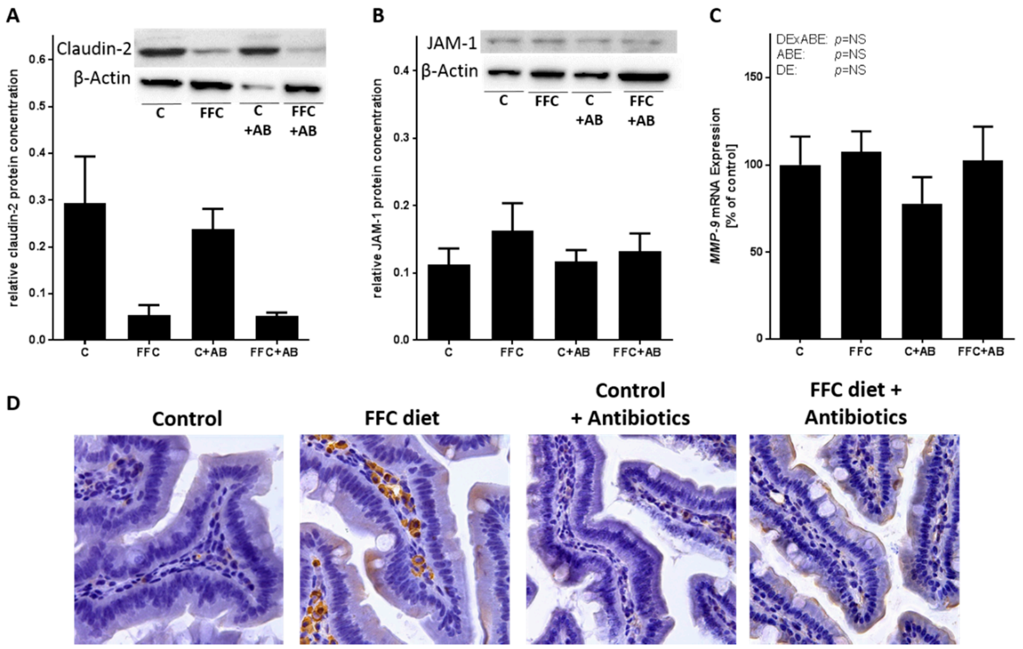

Figure S1. Effect of a short-term feeding of a FFC or control diet ± oral antibiotic treatment on different tight junction proteins and MMPs in small intestine. (**A,B**) Representative pictures and quantitative analysis of claudin-2 and JAM-1 Western blot (n=4), (**C**) *MMP-9* mRNA expression and (**D**) representative pictures of MMP-13 protein staining in small intestine. ABE: antibiotic effect, C: control diet, C+AB: control diet and oral treatment with antibiotics, DE: diet effect, DExABE: interaction between diet and antibiotics, FFC: fat-, fructose- and cholesterol-rich diet, FFC+AB: fat-, fructose- and cholesterol-rich diet and oral treatment with antibiotics, JAM-1: junctional adhesion molecule-1, MMP: matrix metalloproteinase, NS: not significant.
